# fMRI in Non-human Primate: A Review on Factors That Can Affect Interpretation and Dynamic Causal Modeling Application

**DOI:** 10.3389/fnins.2019.00973

**Published:** 2019-09-18

**Authors:** D. Blair Jovellar, Doris J. Doudet

**Affiliations:** ^1^Division of Neurology, Department of Medicine, University of British Columbia, Vancouver, BC, Canada; ^2^Center of Neurology, Hertie Institute for Clinical Brain Research, University Hospital, Tuebingen, Germany

**Keywords:** non-human primate, fMRI, effective connectivity, dynamic causal modeling, image analysis, DCM, anesthesia, BOLD

## Abstract

Dynamic causal modeling (DCM)—a framework for inferring hidden neuronal states from brain activity measurements (e. g., fMRI) and their context-dependent modulation—was developed for human neuroimaging, and has not been optimized for non-human primate (NHP) studies, which are usually done under anesthesia. Animal neuroimaging studies offer the potential to improve effective connectivity modeling using DCM through combining functional imaging with invasive procedures such as *in vivo* optogenetic or electrical stimulation. Employing a Bayesian approach, model parameters are estimated based on prior knowledge of conditions that might be related to neural and BOLD dynamics (e.g., requires empirical knowledge about the range of plausible parameter values). As such, we address the following questions in this review: What factors need to be considered when applying DCM to NHP data? What differences in functional networks, cerebrovascular architecture and physiology exist between human and NHPs that are relevant for DCM application? How do anesthetics affect vascular physiology, BOLD contrast, and neural dynamics—particularly, effective communication within, and between networks? Considering the factors that are relevant for DCM application to NHP neuroimaging, we propose a strategy for modeling effective connectivity under anesthesia using an integrated physiologic-stochastic DCM (IPS-DCM).

## Introduction

Neuroimaging analyses in humans and non-human primates (NHP) have become increasingly sophisticated. One such innovative image analysis technique is dynamic causal modeling (DCM) which has been applied to human fMRI data (Friston et al., 2003; Rowe et al., 2010; Boly et al., 2012; Havlicek et al., 2017; Park et al., 2018; Tak et al., 2018). DCM is a method for inferring hidden neuronal states from brain activity measurements (e.g., fMRI) and their context-dependent modulation (Stephan et al., 2010). Using a Bayesian framework, DCM generates a predicted time serious using a set of differential equations to model neural dynamics. Then, one estimates model parameter by optimally fitting the predicted time series with the observed data. Using DCM, one can test mechanistic hypotheses about how the observed data was generated.

Utilizing a Bayesian approach, model parameters are estimated based on prior knowledge of conditions that might be related to neural and BOLD dynamics (e.g., requires empirical knowledge about the range of plausible parameter values). As such, we discuss: (1) what factors need to be considered when applying DCM to NHP (keeping in mind that it was designed for human fMRI data); and (2) considering said factors, what strategies can one implement when modeling effective connectivity to fMRI data recorded under anesthesia. The latter is an important consideration in NHP fMRI, since most imaging experiments are done under anesthesia and anesthetics have been demonstrated to impart changes in BOLD and neural dynamics, particularly the inhibitory drive (Martin et al., 2006; Masamoto et al., 2007; Moran et al., 2011; Aksenov et al., 2015; Paasonen et al., 2018).

Herein we discuss the following: (1) DCM overview and motivation for NHP data application; (2) comparison of cerebrovascular architecture and functional networks between humans and NHPs; (3) effects of anesthetics on vascular physiology, BOLD contrast, and neural dynamics—focusing on effective communication within and between networks. Lastly, we propose a strategy for modeling effective connectivity under anesthesia using an integrated physiologic-stochastic DCM (IPS-DCM). Taking all these together, we can avoid pitfalls in DCM application in NHP data and facilitate more accurate interpretation of the observed neural dynamics as measured through BOLD fMRI.

## Dynamic Causal Modeling (DCM) in Non-human Primates

### DCM Overview

This section discusses an overview of DCM, including its assumptions, and the motivations for NHP data application. DCM, developed by Friston et al. (2003), is a method that allows estimation of network dynamics and how the dynamic neuronal states give rise to the measured data (Heinzle and Stephan, 2018), as well as how functional coupling is affected by experimental factors. When applied to fMRI data, the BOLD signal is considered a measurable (observed) variable (y) of the underlying neural activity (z) that is not directly measured with fMRI; hence, neural activity is considered a “hidden state variable” (Kahan and Foltynie, 2013). As a measure of effective connectivity (EC), DCM quantifies the directed (causal) influence between regions (Friston, 2009) and considers the rate of change of neural activity over time (z) in response to incoming signals—afferents from other brain regions, experimental manipulation, or both. This is done by creating generative models which are plausible models of how the observed BOLD signal may be generated by those influences.

In DCM, one needs to specify which regions to include in the overall model. As such, DCM follows an analysis that can address which regions in the brain an experimental manipulation induces changes in BOLD response such as a general linear model (GLM) (Stephan et al., 2004). In GLM, time series from each voxel are fitted with an experimental design matrix wherein each condition is specified and the relationship between neural and BOLD response is modeled via a hemodynamic response function (Pernet, 2014). When there are no contrast differences between conditions, there is no motivation to do DCM. Once the relevant regions are identified with GLM, the time series from each region of interest is extracted. After which, one specifies the model architecture—the location of intrinsic connections, driving, and modulatory inputs. Then, one proceeds to estimate the parameters of the generative model.

DCM can offer a more accurate modeling of network dynamics from fMRI data as it considers both the distributed neuronal interactions, and the transformation of the neuronal dynamics to the measured (BOLD) signal (Havlicek et al., 2015). This is accomplished through three fundamental components of the DCM generative model: (1) neuronal (connectivity) model, (2) hemodynamic “Balloon” model, and (3) BOLD signal change equation (Stephan et al., 2007; Havlicek et al., 2015; Friston et al., 2017). A discussion on strategies for modifying the generative model for NHP fMRI under, particularly under anesthesia, will be discussed in a later section.

Each region or node is represented by (differential) neural state equations that provide an abstraction of the summed activity of a neuronal population. The neural state equation is comprised of three parameters that embody: (1) intrinsic connectivity among regions in the absence of input (A matrix); (2) direct influence of extrinsic input on neuronal activity (C matrix); and (3) context-dependent change in connectivity induced by the input (B matrix). Neural dynamics in DCM have been characterized by single-state, two-state, and adaptive two-state equations. The single-state neural equation (Friston et al., 2003) of the classical DCM models synaptic activity of a single excitatory neuronal population in a cortical region—with the rationale that most cortico-cortical connections are excitatory. Two-state (Marreiros et al., 2008) and adaptive two-state (Havlicek et al., 2015) neural equations model both excitatory (glutamatergic) and inhibitory (GABAergic) connections within each region, which makes it a more physiological representation of neural population dynamics. In contrast to the two-state model, the adaptive two-state model includes parameters which allow for adaptation and refractory effects of the neuronal response (Havlicek et al., 2015). These are described in detail in Friston et al. (2003); Marreiros et al. (2008); Havlicek et al. (2015).

The neural model is combined with the hemodynamic model (Friston et al., 2003)—which incorporates the dynamics of neurovascular coupling and the Balloon–Windkessel model (Buxton et al., 1998; Mandeville et al., 1999). This biophysical forward model provides region-specific estimates of the translation of neuronal activity into a predicted BOLD response—as such, hemodynamic states are a function of the neuronal state/s of each region (Friston et al., 2003). The hemodynamic model is comprised of four differential equations that characterize how for each region pre-synaptic activity drives hemodynamic responses—which are mediated by astrocytic Ca^2+^ signaling whose endfeet release vasodilatory metabolites (MacVicar and Newman, 2015). This vasodilatory signal is subject to auto-regulatory feedback (Attwell and Iadecola, 2002; Friston et al., 2003) and blood flow changes proportionately to the vasodilatory signal which leads to an increase in blood volume with concomitant decrease in deoxyhemoglobin (Stephan et al., 2004).

The output signal is defined by the BOLD signal change equation—which links blood volume and deoxyhemoglobin content to the BOLD signal change (Stephan et al., 2007). The resulting BOLD signal depends on the depends on the relative contributions of intra- and extravascular signals (Buxton et al., 2004; Friston et al., 2017) and follows the flow dynamics with a delay of ~1 s (Stephan et al., 2004). The BOLD signal change equation completes the DCM generative model.

In standard DCM, the parameters of the full forward model (combined neural and hemodynamic models) is estimated from the measured BOLD data using is estimated iteratively from the measured BOLD data using Variational Bayesian (VB) algorithm (Friston et al., 2007) to produce probabilistic estimates of the expected value of each parameter given the data. Details of the parameter estimation process are beyond the scope of this review and the reader is referred to Friston et al. (2002). The objective of the estimation process is to generate a predicted signal that closely matches the observed BOLD data (Kahan and Foltynie, 2013). From the DCM parameter estimates, one can then quantify and make inferences about connection strength and direction between regions based on prior assumptions about connectivity architecture.

Finally, Bayesian model selection (BMS) is done to compare a series of models that represent different prior hypotheses of connectivity architecture to examine which of the competing models most likely generated the observed data. This is done by comparing the evidence for each model. Model evidence—the normalization constant for the product of the likelihood of the data and prior probability of the parameters—is approximated using Laplace approximation in standard DCM (Stephan et al., 2007). This yields the log-evidence for each model, characterized as the conditional probability (posterior probability) of competing models given the observed data (Kahan and Foltynie, 2013). The optimal model is one that represents the best compromise between model fit (accuracy) and complexity (characterized by the number of free parameters in the model) (Stephan et al., 2007)—it is the best fitting, yet, most parsimonious model. As such, this model is more generalizable.

### DCM Assumptions

There are three main assumptions in DCM: (1) deterministic assumption on the inputs that enter the system; (2) Gaussian assumption on the posterior density; and (3) assumption of equal detection of BOLD signal changes throughout the brain. Firstly, in classical DCM, all processes in the system are considered deterministic such that it is presumed that neural dynamics in a region is entirely due to incoming afferents from other regions and/ or experimental inputs. This assumption is not always tenable in NHP fMRI studies which are mostly done under anesthesia—this restricts task-based imaging to simpler visual/ auditory experiments and many imaging experiments are task-free. However, extensions of DCM in humans have allowed its application in resting state studies: (1) using classical DCM, one can stimulate nodes with fluctuations of specific frequencies (Di and Biswal, 2014); (2) modeling random fluctuations in neural dynamics explicitly using stochastic DCM (Li et al., 2011; Daunizeau et al., 2012); or (3) estimating the spectral density of neuronal fluctuations such that effective connectivity of hidden neuronal states is a function of observed functional connectivity from hemodynamic responses (Friston et al., 2014; Park et al., 2018). Secondly, parameter estimates of the generative model are assumed to be Gaussian—i.e., that the values are normally distributed. This may be a concern when using two-state DCM which use exponentiated scale parameters that introduce positivity constraints as these values likely do not have a normal distribution (Hillebrandt et al., 2014). Lastly, DCM rests on the assumption that BOLD signal detection is equally sensitive across brain regions. This was validated by Friston et al. (2003) by simulating region-specific dropout wherein they found that DCM does not accommodate substantial signal dropout (e.g., 50%). In the absence of profound dropout, DCM is robust to regional variations in sensitivity to BOLD signal changes.

### Why Apply DCM to NHP FMRI?

The motivations for applying DCM to NHP data are founded on the prospective to make DCM models more accurate with animal experiments that are not yet feasible or are considered unethical in humans, as well as on being a more physiologically-informed characterization of network dynamics vs. previous effective connectivity models.

Animal imaging data and experiments possess the potential to make DCM models more accurate. Invasive procedures such as *in vivo* optogenetic and electrical stimulation can be combined with functional imaging to examine how disruptions at the microscale can affect whole brain network dynamics. Additionally, one can also investigate the temporal evolution of the effects of drugs (e.g., administered intravenously or through gas inhalation). Moreover, advances in high-field fMRI at the sub-millimeter scale, allows imaging and modeling at laminar resolutions (Heinzle et al., 2016; Friston et al., 2017).

In order to accurately disentangle neuronal- and hemo-dynamics, experimental manipulation can provide necessary constraints to estimate hemodynamic parameters, which may explain discrepancies in response shape between neuronal and BOLD response. This can be addressed by using multi-modal recordings (e.g., simultaneous measurements of CBF, CBV, and BOLD), which is often more feasible in animal studies, including NHP. For instance, Havlicek et al. (2017) showed that combined analysis of BOLD and CBF data yields more robust effective connectivity estimates.

Furthermore, DCM was originally developed specifically for fMRI data (Friston et al., 2003) which gives it an edge over other models such as Granger causality and structural equation modeling (SEM) that were initially applied in the fields of economics, psychology, and genetics (Wright, 1920; Granger, 1969). Compared with previous EC measures, DCM allows a more physiologically-informed characterization of network dynamics as it incorporates a hemodynamic model that has previously been experimentally validated (Buxton et al., 1998; Friston et al., 2003). On the other hand, the disadvantages of Granger causality in fMRI application is plentiful. Using four different algorithms, Smith et al. (2011) demonstrated that Granger causality exhibits poor (<20%) sensitivity in connection link detection, false positive identification and directionality estimation (Smith et al., 2011). Moreover, Witt and Meyerand (2009) found that Granger causality has poor sensitivity and specificity (close to chance levels) when modeling data including intrinsic variance from trimmed time series. While DCM is computationally costly, its ability to model non-linear and dynamic neuronal interactions (Bielczyk et al., 2019), as well as both unidirectional and bidirectional connections (Vaudano et al., 2013; Buijink et al., 2015) give a more accurate picture of underlying neuronal activity. While originally designed for task fMRI, recent developments such as stochastic or spectral DCM allow modeling of resting state fMRI (Li et al., 2011; Daunizeau et al., 2012; Friston et al., 2014; Park et al., 2018). Classical DCM (Friston et al., 2003) pose restrictions on network size as increasing the number of nodes considerably increases computational time (Bielczyk et al., 2019). However, ensuing extensions allow exploratory studies involving larger networks such as spectral DCM (Friston, 2011) for resting-state fMRI and DCM with sparsity constraints for task fMRI (Frässle et al., 2018). The many advantages of DCM over previous effective connectivity measures and the ability to combine invasive procedures in neuroimaging make DCM application to NHP data an exciting endeavor and may offer the possibility to improve the accuracy of causal models. A summary of the advantages and disadvantages of the commonly used effective connectivity models (structural equation modeling, Granger causality, transfer entropy, and dynamic causal modeling) is outlined in Table 1.

**Table 1:** Comparison of commonly used effective connectivity models.

	**Pros**	**Cons**
Structural equation modeling	* Can detect excitatory and inhibitory connections and connection strength (Bielczyk et al., 2019) * Sensitivity to small changes in path weight values due to large dynamic range (Witt and Meyerand, 2009)	* Difficulty in estimating reciprocal and cyclic connections (physiologically, reciprocal connections are ubiquitous in the brain) (Friston, 2011) * May not be as suitable to event-related design due to the assumption that random fluctuations change very slowly in relation to neuronal dynamics such that neuronal dynamics has already reached steady-state at the time of recording (Friston, 2011) * May be inappropriate in the context of disease or pharmacologic experiments that can affect hemodynamic response function (Rowe et al., 2010)
Granger causality	* Can detect excitatory and inhibitory connections and connection strength (Bielczyk et al., 2019) * Yields bidirectional connections (Bielczyk et al., 2019) * Results can be mapped onto the brain similar to fMRI (Goebel et al., 2003; Roebroeck et al., 2005; Witt and Meyerand, 2009)	* Poor sensitivity and specificty (close to chance levels) when modeling data including intrinsic variance from trimmed time series (Witt and Meyerand, 2009) * Assumption of signal stationarity (Seth et al., 2015) * Restriction on network size–the number of nodes divided by the number of shifts can never exceed the number of time points (Bielczyk et al., 2019) * Markovian assumption that random terms in the vector autoregression model are serially independent may not hold when the terms become temporally correlated upon converting from continuous to discrete time formulations (Friston, 2011) * The spatial distribution of GC has been associated with the Circle of Willis and identifies major arteries and veins as causal hubs (Webb et al., 2013) * Assumption of uniform hemodynamic response function across regions may elicit spurious causal relationship when one region has faster hemodynamic activity–the temporal precedence of the peak in one region may be mistaken for Granger causing the other (Bielczyk et al., 2019) * fMRI temporal resolution may be too slow for accurate depiction of neural dynamics using Granger causality (Witt and Meyerand, 2009) * Poor (<20%) sensitivity in connection link detection, false positive identification and directionality estimation (Smith et al., 2011)
Transfer entropy	* Can detect excitatory and inhibitory connections and connection strength (Bielczyk et al., 2019) * Captures linear and non-linear interactions between nodes (Bielczyk et al., 2019) * Computationally cost-efficient (Vicente et al., 2011)	* Restriction on network size–the number of nodes divided by the number of shifts can never exceed the number of time points (Bielczyk et al., 2019) * Imposes a time-lag in the inference procedure with similar disadvantages as Granger Causality in fMRI application (Schreiber, 2000)
Dynamic causal modeling	* Developed specifically for fMRI (Friston et al., 2003) data and incorporates a biologically-informed model of BOLD dynamics (Buxton et al., 1998), unlike other models Granger causality and SEM were originally applied in the fields of economics, psychology, and genetics (Wright, 1920; Granger, 1969) * Can detect excitatory and inhibitory connections and connection strength (Bielczyk et al., 2019) * Can model both unidirectional and bidirectional connections (Vaudano et al., 2013; Buijink et al., 2015) * Models nonlinear and dynamic neuronal interactions (Bielczyk et al., 2019) * Classical DCM is suitable for event-related designs (Rowe et al., 2010) * Stochastic or spectral DCM is suitable for resting state studies (Li et al., 2011; Daunizeau et al., 2012; Friston et al., 2014; Park et al., 2018) * For exploratory studies involving larger networks, spectral DCM (Friston, 2011) can be applied for resting-state fMRI while DCM with sparsity constraints can be applied for task fMRI (Frässle et al., 2018) * High reproducibility (Rowe et al., 2010; Schuyler et al., 2010; Bernal-Casas et al., 2013; Tak et al., 2018)	* Computationally-expensive (Bielczyk et al., 2019) * Restriction on network size (using classical DCM)—increasing the number of nodes considerably increases computational time (Bielczyk et al., 2019) * Depends on prior assumptions on connectivity architecture (Friston et al., 2003) * Assumes all models are equally likely (even implausible models) (Lohmann et al., 2012); hence, substantial knowledge is needed to define all plausible causal connections between nodes

## Considerations Specific to DCM Application in NHP

### Functional Networks and Cerebrovascular Architecture: Human vs. NHP

#### Cerebrovascular Architecture and Physiology

The cerebral vasculature of NHP is largely similar to humans in terms of the architecture of superficial pial vessels and intracortical vessels (Duvernoy et al., 1981; Weber et al., 2008). Similarly found in humans (Duvernoy et al., 1981), large vessels are found on the surface of the macaque brain (Weber et al., 2008). The density of superficial vessels have been found to vary across cortical regions—the occipital lobe surface is highly vascularized compared to the less dense vascularization at the top of the hemispheres near the interhemispheric fissure (Scharrer, 1960; Duvernoy et al., 1981). In terms of vessel diameter, pial veins generally have a larger diameter than arteries— central veins have an average diameter of 280–380 μm and peripheral veins average 130 μm, while central arteries have a diameter of 260–280 μm and peripheral arteries average 150–180 μm (Duvernoy et al., 1981; Guibert et al., 2010). As for intracortical vessels, the laminar distribution of vessels in NHP are similar to that found in humans. Studying the primate visual cortex, Bell and Ball (1985) found a high density of cortical vessels in layer IVC that ends at the boundary between primary and secondary visual cortices—akin to the vascular distribution in humans.

The fluctuations in deoxyhemoglobin concentration detected by the BOLD contrast depends on the combined changes in cerebral blood volume (CBV), cerebral blood flow (CBF), and cerebral metabolic rate of oxygen (CMRO2) (Buxton et al., 2014). In general, the values for each hemodynamic component is comparable between humans and NHP. Van Aken et al. (1986) found CBF at 48 ± 4 mL/100 g/min in baboons. In humans, Olsen et al. (1994) demonstrated 51 mL/100 g/min, while Ito et al. (2004) found 69.8 ± 15.4 mL/100 mL/min CBF values. CBV ranged from 3.5 to 4.7 ml/100 g of brain tissue in the macaque (Phelps et al., 1973; Grubb et al., 1974; Eichling et al., 1975) while CBV = 3.8 ± 0.7 ml/100 ml^−1^ (Ito et al., 2004). As for CMRO2, Van Aken et al. (1986) obtained 3.64 ± 1 (ml/100 g/min) in baboons and Olsen et al. (1994) found 3.5 (ml/100 g/min) in humans. The values for CBF and CMRO2 are modulated by anesthetics and this will be discussed in an ensuing section.

#### Functional Differences

Resting-state fMRI studies comparing human and NHP connectivity have revealed three main differences. First, there are differences in specific connectivity patterns that may potentially indicate cognitive specializations in humans. For example, comparing the organization of the dorsal frontal cortex between humans and macaques (Sallet et al., 2013), found a high degree of similarity in functional coupling patterns between the medial frontal cortex and other regions (i.e., frontal pole, medial prefrontal, and dorsal frontal convexity) in both. However, certain regions in the dorsolateral prefrontal cortex (areas 9/46) were coupled with the superior and medial parietal cortex in humans but not in macaques. Further, Mars et al. (2011) demonstrated resting-state functional connections between anterior prefrontal cortex and central inferior parietal lobule (IPL) in humans which are not found in macaques. Second, there may be species-specific differences in cortical hub distribution. Upon mapping putative hubs in humans, chimpanzees, and macaques, Li L. et al. (2013) demonstrated the ventrolateral prefrontal, medial parietal and retrosplenial cortices are hubs across three species. In contrast, medial prefrontal, inferior parietal, and V1 cortices were hubs in macaques and chimpanzees and not in humans. Additionally, superior parietal and medial premotor cortices were hubs in humans and not in the NHPs. Third, though it has been demonstrated that there are 11 functionally correspondent networks in both humans and macaques, three networks were found in humans that are missing in the latter (Mantini et al., 2013). While sensory-motor, attention, language, and default mode networks are evolutionarily conserved, two lateralized fronto-parietal networks are unique to humans (Van Essen and Dierker, 2007). These have been implicated in general intelligence (Duncan et al., 2000), abstract reasoning (Dehaene et al., 2003), and tool use, particularly retrieving and planning transitive actions for subsequent hand motor behavior (Frey, 2008). A third human-specific network includes the anterior insula and dorsal anterior cingulate cortex—both putatively involved in empathy (Singer and Lamm, 2009).

### The Anesthetized Brain

#### Anesthetic Effect on Vascular Physiology and BOLD Contrast

Anesthetics have been found to impart changes in cerebrovascular function and the BOLD signal. As these are incorporated in the DCM generative model, it is important to examine how these are affected in the anesthetized brain, particularly since most NHP neuroimaging is done under anesthesia.

There are conflicting results in the regional distribution of anesthetic-induced changes in vascular physiology. Li C.-X. et al. (2013) examined the dose-dependent effect of isoflurane on regional CBF of cortical and subcortical structures in macaques. They found that high isoflurane concentrations (i.e., 1.5%) resulted in global CBF increase which was most evident in subcortical structures—specifically in the thalamus and cerebellum in macaques. Interestingly, under the 0.75–1.5% isoflurane maintenance doses, there were no observable CBF changes in cortical regions (i.e., anterior cingulated cortex, motor cortex, medial prefrontal cortex) and the caudate. These indicate that while CBF auto-regulation is intact in cortical regions and the caudate under isoflurane maintenance dose, it is impaired in the thalamus and cerebellum, and suggest that subcortical structures contribute the most to the increase in global CBF. On the other hand, Långsjö et al. (2005) demonstrated that ketamine increased whole brain CBF in humans—with the highest increase in the anterior cingulate. They found that CMRO2 increased only in the frontal cortex while glucose metabolism increased only the in the thalamus. Långsjö et al. (2005) assert that this indicates that majority of the increases in CBF most likely do not indicate neuronal activation. Another study by Van Aken et al. (1986) showed that the impact of isoflurane on CBF was biphasic—low levels (0.5 ± 0.35 vol%) resulted in vasoconstriction and decreased CBF while higher concentrations (0.95 ± 0.7 vol% and 1.4 ±1 vol%) caused vasodilation and increased CBF to baseline levels (no anesthesia). While they found that the effect of isoflurane on CBF was biphasic, CMRO2 continually decreased in a dose-dependent manner. The disparity in the findings of these studies may be attributed to methodological differences in anesthetic (isoflurane vs. ketamine), species (human vs. primate), and imaging modality (arterial spin labeling MRI vs. PET). Nonetheless, the results of these studies indicate anesthetic-related disruption of CBF-metabolism coupling characterized by more widespread CBF increases with minor changes in CMRO2 and glucose metabolism, with dose-related variabilities in response.

As for the impact of anesthetics on BOLD contrast, anesthetics have been demonstrated to cause changes in the spectral components and decrease the activated area and magnitude of the signal, as well as change the hemodynamic response temporal structure (Martin et al., 2006; Aksenov et al., 2015; Paasonen et al., 2018). Paasonen et al. (2018) found that awake mice exhibit higher spectral BOLD power at a wide frequency range. In contrast, they found that all six anesthetic conditions studied strongly suppressed power and BOLD fluctuations occurred at narrower frequency ranges, which potentially reflect more homogeneous activity. This was a predictable outcome as by definition, anesthetics exert an inhibitory effect on brain function. Aksenov et al. (2015) suggested that enhanced GABA-A receptor inhibition and diminished afferent input reduces the spread of stimulus-related activity, which results in more focal activation and decreased BOLD response area. Further, they surmised that decreased BOLD response magnitude reflects reduced thalamic input and intra-cortical processing associated with decreased neuronal excitation. A decrease in hemodynamic response magnitude under anesthesia has also been demonstrated in previous studies employing optical imaging spectroscopy (OIS) (Berwick et al., 2002). Aside from decreased duration of the BOLD signal reported by Aksenov et al. (2015), Martin et al. (2006) found that anesthesia increased hemodynamic response latency (~2 s awake vs. 4 s anesthetized).

#### Anesthetic Impact on Effective Communication Within and Between Networks

It is now widely acknowledged that anesthetics modulate communication within and between networks. Anesthetic-induced perturbations in effective connectivity have been observed across multiple neuroimaging techniques, species (humans and rodent models), and anesthetics. In particular, effective connectivity changes have been demonstrated in thalamo-cortical, cortico-thalamic, and cortico-cortical (both association and sensory cortices) connections. Under propofol, ketamine, or isoflurane, selective disruption of frontal to parietal feedback has been widely replicated in human subjects and rats (Imas et al., 2005; Lee et al., 2009; Boly et al., 2012)—particularly gamma frequencies (50 Hz) (Imas et al., 2005). In addition, Gómez et al. (2013) found that propofol decreased feedback from middle frontal gyrus to superior temporal gyrus. As for sensory-motor regions, multiple studies found impaired effective connectivity under anesthesia. White and Alkire (2003) demonstrated impaired effective drive from the supplementary motor area (SMA) to the primary motor (M1) cortex in humans, while Kang et al. (2016) showed abolished causal flow of 7–12 Hz activity from primary sensory (S1) to M1 and ventrobasal thalamic nucleus in mice. Decreased feedforward and feedback connections were found within auditory cortical regions (Heschl's gyrus and superior temporal gyrus) in humans under propofol (Gómez et al., 2013). Pertaining to anesthetic-related changes in thalamocortical effective connectivity, the results are more heterogeneous. Causal flow from the thalamus to the anterior cingulate and posterior parietal cortices was intact under propofol (Boly et al., 2012). On the other hand, effective connectivity from the thalamus to the SMA was impaired under halothane or isoflurane (White and Alkire, 2003). However, effective thalamic drive to M1 has shown variable results—remaining unchanged under ketamine in mice (Kang et al., 2016) compared to the awake state, while decreasing under halothane or isoflurane in humans (White and Alkire, 2003). This discrepancy may indicate species-specific differences in thalamic-motor connectivity in response to anesthetics, differences in regional distribution of different anesthetics (ketamine vs. halothane or isoflurane), or both. Altogether, these findings indicate that anesthetics induce: (1) disruption of higher-order information processing; and (2) reduced capacity for sensory-motor integration.

### Relevance to DCM in NHP

For DCM to generate reliable neuronal signal estimates, having an accurate model of the hemodynamic response is crucial; thus, it is important to assess whether differences in cerebrovascular architecture and physiology in NHP—factors that contribute to the BOLD contrast—may affect priors on the biophysical parameters of the model.

The cerebrovascular architecture and laminar distribution of blood vessels in NHPs are similar to that of humans. The fMRI BOLD signal indicates changes in deoxyhemoglobin and the signal is sensitive to fluctuations in venous blood volumes. The cerebral vasculature of NHP is largely similar to humans in terms of the architecture of superficial pial vessels and intracortical vessels (Duvernoy et al., 1981; Weber et al., 2008; Adams et al., 2015). By extension, the BOLD signal-to-noise ratio in superficial to deep brain areas is expected to have similar distribution in both.

More importantly, the BOLD signal is a function of CBV, CBF, and CMRO2—which reflects fluctuations in deoxyhemoglobin content (Buxton et al., 2014), and DCM includes priors on these hemodynamic parameters. Since the CBV, CBF, and CMRO2 in NHP are comparable to human values (Phelps et al., 1973; Grubb et al., 1974; Eichling et al., 1975; Van Aken et al., 1986; Olsen et al., 1994), it is tempting to presume that one can proceed with applying DCM in NHP without adjustments on the biophysical parameters if one solely considers cerebrovascular architecture and physiology.

Notably, anesthetics such as isoflurane and ketamine have been found to increase blood flow (Van Aken and van Hemelrijck, 1991; Långsjö et al., 2005; Slupe and Kirsch, 2018), which is relevant in primate neuroimaging as it is usually done under anesthesia. The decision on how to proceed with DCM in the anesthetized brain depends on the research question. Upon comparing eight different hemodynamic models by modifying the coefficients of the BOLD signal change equation, Stephan et al. (2007) assert that variations in the hemodynamic model are relatively inconsequential when making inferences on underlying neuronal dynamics and their causal influences. This is due to the relative independence of the parameters of the neural state equation—characterizing intrinsic connectivity and their context-dependent modulation (A and B matrices)—from the amplitude of the hemodynamic response and the direct influence of extrinsic inputs on neuronal activity (matrix C). On the other hand, if the research question focuses on regional variations in hemodynamic parameters (e.g., in healthy vs. clinical populations), then preliminary tests need to be performed to determine the effect of variations on the priors of the biophysical model—related to anesthetic influence. For instance, if the experimenter can identify the baseline CBF and CBV using multi-modal recordings, the mean transit time (i.e., baseline CBV/baseline CBF)—which scales the CBV and deoxyhemoglobin changes in the hemodynamic model—could be directly calculated. Other strategies for modifying the hemodynamic model are discussed in the last paragraph of the next section on IPS-DCM parameters.

Moreover, caution is necessary when interpreting results if the model includes regions or networks in which effective connectivity may be modulated by anesthetics. Regions most affected by anesthetics are: (1) fronto-parietal (Ku et al., 2011; Boly et al., 2012; Kim et al., 2017); (2) sensory-motor (i.e., S1 to M1, SMA to M1, auditory cortical regions) (White and Alkire, 2003; Gómez et al., 2013); and (3) thalamocortical networks specifically involving somatosensory and motor function (White and Alkire, 2003; Kang et al., 2016). Since effective drive of these networks are reduced or abolished by anesthetics, it may result in underestimation of the effect of interventions (e.g., medications, brain stimulation) on connectivity within and between affected networks. It may also result in the overestimation of the modulatory effects of different interventions if aimed at reducing hyperconnectivity.

Additionally, anesthetics can modulate neural dynamics by altering inhibitory drive, neural refractory period, and cortical adaptation (Masamoto et al., 2007; Moran et al., 2011). These artifacts can be addressed by using an adaptive two-state neural connectivity model (Havlicek et al., 2015). This and other strategies for applying DCM in NHP under anesthesia are discussed in the succeeding section.

## Integrated Physiologic-Stochastic DCM: Modeling Effective Connectivity Under Anesthesia

### IPS-DCM Parameters

An important factor to consider upon implementing DCM in the anesthetized NHP is the type of DCM to use. Anesthetics can decrease BOLD signal-to-noise ratio and artificially lower spontaneous fluctuations and activity correlations (Hutchison et al., 2014). In addition to dose-dependent linear decrease in glutamatergic excitatory postsynaptic potentials (EPSPs) together with non-linear increase (saturating) in GABAergic inhibitory postsynaptic potentials (IPSPs) (increasing local inhibitory drive) (Moran et al., 2011), anesthetics modulate the neural refractory period and cortical adaptation with varying degrees depending on anesthetic type and dose (Masamoto et al., 2007). To overcome these limitations, we propose the combined use of two DCM extensions: stochastic DCM (Li et al., 2011) and physiologically-informed DCM (P-DCM) which has an adaptive two-state neural connectivity equation (Havlicek et al., 2015).

Stochastic DCM models fluctuations in hidden states (e.g., neuronal or hemodynamic states) that are due to endogenous (autonomous) dynamics, not exogenous experimental inputs (Li et al., 2011). By accounting for noise in the model using stochastic DCM, the risk of under-estimating effective connectivity parameters can be reduced (Gómez et al., 2013). Additionally, stochastic DCM is more useful when there are non-linear interactions among hidden states, such as the non-linear increase in GABAergic inhibitory drive (Moran et al., 2011; Daunizeau et al., 2012). Finally, Li et al. (2011) demonstrated that stochastic DCM using the generalized filtering (GF) inversion method showed higher sensitivity in detecting group differences compared to the variational Bayesian (VB) algorithm (Friston et al., 2007) of standard deterministic DCM.

Two-state (Marreiros et al., 2008) and adaptive two-state (Havlicek et al., 2015) neural equations model both excitatory (glutamatergic) and inhibitory (GABAergic) connections within each region, which makes it a more physiological representation of neural population dynamics. In contrast to the two-state model, the adaptive two-state model includes parameters which allow for adaptation and refractory effects of the neuronal response (Havlicek et al., 2015). This extension is relevant since anesthetics induce changes to the neural refractory period and cortical adaptation (Ogawa et al., 1992; Masamoto et al., 2007). Furthermore, explicitly modeling both excitatory and inhibitory signals may help capture the potentiation of GABAergic inhibition under anesthetics (Moran et al., 2011). This may be elucidated in the dynamics of the inhibitory subpopulation or the interaction between excitatory and inhibitory subpopulations (Gómez et al., 2013).

The adaptive two-state DCM is the neuronal connectivity component of P-DCM developed by Havlicek et al. (2015). The parameters that incorporate adaptation and refractory effects to neuronal response are: (1) the inhibitory gain factor λ, which modulates the amplitude and temporal smoothness of the inhibitory activity in relation to the excitatory drive, and (2) the inhibitory–excitatory connection μ which reflects the temporary imbalance in temporal smoothness between excitatory and inhibitory activity that can result in neuronal adaptation.

The second modification in P-DCM involves the hemodynamic model. The changes are 2-fold: (1) modeling feedforward neurovascular coupling (vs. feedback NVC in classical DCM); and (2) incorporating a viscoelastic effect in the Balloon model. Experiments by Lindauer et al. (2010) and Powers et al. (1996) show that manipulating oxygen and glucose levels in the blood do not regulate blood flow as per negative feedback hypotheses (Attwell et al., 2010; Havlicek et al., 2015). Additionally, hypercapnia experiments demonstrated that higher baseline CBF has minimal impact on the absolute stimulus-induced CBF change (while relative CBF became smaller) (Li et al., 2000; Brown et al., 2003; Zappe et al., 2008). This latter point is pertinent to DCM application in NHP since isoflurane—the most commonly used anesthetic in animal experiments—is a potent vasodilator and causes higher baseline CBF that may be uncoupled from cerebral energy metabolism (Van Aken and van Hemelrijck, 1991). Thus, feedforward neurovascular coupling is more relevant in NHP image analysis, particularly under anesthesia. On the other hand, the viscoelastic component in the hemodynamic model was added to account for transient responses (i.e., BOLD post-stimulus undershoot and overshoot) outside of the steady-state relationship between CBF and CBV, described by the power law where α = 0.38 (Grubb et al., 1974). The duration of the transient adjustment period is regulated by viscoelastic time constant τ –which allows for variations in outflow curve during balloon inflation and deflation, corresponding to BOLD response overshoot and undershoot (Buxton et al., 1998, 2004). The BOLD transients are then presumed to reflect both neuronal post-stimulus deactivation and vascular uncoupling related to slow recovery of venous CBV (Havlicek et al., 2015).

Lastly, the BOLD signal change equation was modified to accommodate different magnetic field strengths. k1, k2, and k3 are parameters that reflect baseline physiological properties of brain tissue and acquisition parameters and have been adjusted to depend on different magnetic field strengths. Moreover, they also suggest revised values for ε (ratio of intra—and extravascular signal) and r0 (regression slope of changes in intra-vascular signal relaxation rate with changes in oxygen saturation) depending on acquisition sequence (gradient echo vs. spin echo) (Uludag et al., 2009; Havlicek et al., 2015).

**Figure 1:**
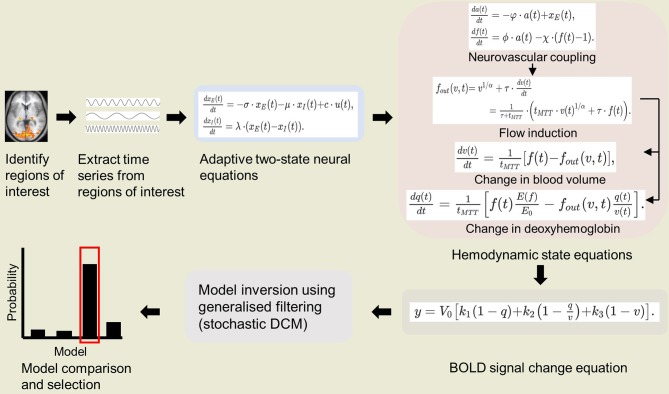
Schematic of integrated physiologic-stochastic DCM (IPS-DCM). After identifying and extracting the time series from regions of interest, model parameters are then estimated using P-DCM equations (Havlicek et al., 2015). P-DCM incorporates: (1) an adaptive two-state neuronal model that allows adaptation and refractory effects to neuronal response; (2) a hemodynamic model that implements feedforward neurovascular coupling and a viscoelastic effect on the Balloon model; (3) a BOLD signal change equation that accounts for magnetic field differences. The model inversion is done using generalized filtering (stochastic DCM) (Li et al., 2011). Lastly, one proceeds to model comparison and selection of the winning model.

As stated above, we propose to combine P-DCM with stochastic DCM. Since stochastic DCM uses the same biophysical forward model as classical DCM, the changes in biophysical parameters in P-DCM can be integrated in the stochastic extension (Havlicek et al., 2015). Thus, in this application of DCM on NHP fMRI, we suggest estimating the full forward model using the biophysical parameters of P-DCM applying the generalized filtering (GF) inversion method of stochastic DCM (Li et al., 2011). A schematic of IPS-DCM is illustrated in Figure 1. A salient consideration here is that stochastic DCM tends to emphasize neuronal dynamics over hemodynamics (Reviewer 2 comment). Nonetheless, as aforementioned, stochastic DCM is a more accurate model when there are non-linear interactions among hidden states, such as the non-linear increase in GABAergic inhibitory drive (Moran et al., 2011; Daunizeau et al., 2012) which is relevant when imaging under anesthesia. For NHP neuroimaging under anesthesia, this is useful for task-based imaging with simple visual/ auditory experiments, and even more so for resting state studies. Therefore, in order to more accurately estimate both neuronal and hemodynamic changes, future extensions of P-DCM (which is so far formulated and applied as a deterministic model) to fully stochastic or partially stochastic (stochastic neuronal model and deterministic hemodynamic model) may be relevant.

With this integrated physiologic-stochastic DCM, one can examine anesthetic impact on effective connectivity in NHP. This can be done by first building generative models of fMRI done pre and post anesthesia. Then, Bayesian model comparison allows one to examine which model best explains the changes in effective connectivity between awake and anesthetized states.

An important consideration here is that anesthetics such as isoflurane and ketamine increase baseline CBF due to increased vasodilation (Van Aken and van Hemelrijck, 1991; Långsjö et al., 2005; Slupe and Kirsch, 2018). It is also a common observation that increased baseline CBF results in smaller relative CBF change (Li et al., 2000; Cohen et al., 2002; Zappe et al., 2008). While current DCMs model relative CBF changes (Friston et al., 2003; Havlicek et al., 2015), the baseline CBF is not directly expressed. Thus, in order to account for the effect that anesthetics have on relative change in CBF, the equation modeling CBF change in DCM models could be further modified to reflect also baseline CBF values. It would be then also beneficial to consider multi-modal data that measure both CBF and BOLD signals (Havlicek et al., 2017). Even if CBF is not measured directly, it is helpful to have baseline CBF as a parameter. Additionally, as mentioned above, anesthesia can the increase latency of CBF response (also later reflected in BOLD response), e.g., from ~2 to ~4 s (Martin et al., 2006). Latency of CBF response can be controlled in DCM models. For example, Havlicek et al. (2017) accounted for differences between latency of positive and negative CBF responses measured in anesthesized NHP by optimizing parameter χ in the feedforward neurovascular coupling of the P-DCM, while other parameters (ϕ, φ) could remain fixed. These considerations could permit more physiologically accurate evaluation of the effect of pre- and post-anesthesia on effective connectivity, as modeled using IPS-DCM.

### Comparison With Other DCM Applications Under Anesthesia

DCM has been applied in two other studies examining connectivity changes under propofol-induced loss of consciousness in fronto-parietal (Boly et al., 2012) and auditory (Gómez et al., 2013) networks. Boly et al. (2012) applied DCM for steady-state responses (SSR) and used neural mass models for each region of the fronto-parietal network (plus thalamic source)—three subpopulations in cortical regions (one excitatory and two inhibitor) and two for the thalamic source (excitatory relay cells and inhibitory reticular cells). On the other hand, Gómez et al. (2013) utilized combined stochastic (Daunizeau et al., 2009) and two-state (Marreiros et al., 2008) DCM to model one frontal and two temporal (auditory) cortical regions. In this section, we discuss the main caveats of both studies and how IPS-DCM may address said limitations.

The main weakness of both studies is the steady-state assumption of both DCMs which may not hold under anesthesia. Boly et al. (2012) found in initial increase in beta and gamma rhythms while delta to alpha frequencies continually increased as loss of consciousness emerged and acknowledge that this may reflect neuronal up and down states. Gómez et al. (2013) asserts that they maintained steady-state by keeping the anesthetic dose fixed after Ramsay evaluation during fMRI recordings. However, Yeom et al. (2017) demonstrated that even when patient-controlled anesthetic levels (propofol and midazolam) were unchanged once consciousness was lost, over time, there was gradually increasing power in frequencies <15 Hz together with decreasing power at >15 Hz. Increased delta and alpha power were most evident in frontal and parieto-occipital regions. Thus, the steady-state assumption of both DCMs may not hold in both studies with anesthetics.

The combined use of the adaptive two-state DCM by Havlicek et al. (2015) and stochastic DCM by Li et al. (2011) offers a number of advantages over the previous two DCM applications. Li et al. (2011) demonstrated that the generalized filtering inversion method in this DCM extension provides better effective connectivity estimates and higher sensitivity to detecting group differences than the expectation maximization (EM) or dynamic expectation maximization (DEM) of classical and variational Bayes stochastic DCM (i.e., it detected two additional connections exhibiting group differences).

As for the neuronal model, Havlicek et al. (2015) performed simulations comparing standard two-state DCM and P-DCM (adaptive two-state) and showed time courses of responses to either 1 s or 30 s stimulation in one region, as well as connectivity dynamics of a three-region network in response to a 30 s stimulation. They demonstrated that with standard two-state DCM, varying the neuronal post-stimulus deactivation does not translate to the BOLD undershoot, while P-DCM accurately models post-stimulus neuronal deactivation, both in response to 1 s or 30 s stimulation. This ability also holds even when CBV and CBF are uncoupled—the BOLD post-stimulus undershoot is stronger than the CBF response which resembles experimental results (Chen and Pike, 2009).

Pertaining to the connectivity dynamics of the simulated neuronal network, standard two-state DCM was unable to capture decreased neuronal activity below baseline—which suggests poor signal variance when both activations and deactivations are in the measured BOLD data. Meanwhile, P-DCM effectively expresses transients in neuronal and BOLD responses during faster and slower neuronal dynamics. This holds in both positive and negative responses (activation and deactivation).

## Conclusion

In summary, the most important factors to consider when applying DCM in NHP under anesthesia are cerebrovascular physiology as well as anesthetic-induced changes in neural and BOLD response dynamics. Moreover, caution is necessary when interpreting results if the model includes regions or networks in which effective connectivity may be modulated by anesthetics such as: (1) fronto-parietal; (2) sensory-motor (i.e., S1–M1, SMA–M1, auditory cortical regions); and (3) thalamocortical networks specifically involving somatosensory and motor function.

The integrated P-DCM (Havlicek et al., 2015) and stochastic DCM (Li et al., 2011) (IPS-DCM) aims to address the modulatory effects of anesthetics on neural activity and the BOLD response such as changes in inhibitory drive, neural refractory period, and cortical adaptation (Masamoto et al., 2007; Moran et al., 2011). This is accomplished through: (1) an adaptive two-state neuronal model that incorporates adaptation and refractory effects to neuronal response; (2) a hemodynamic model that incorporates feedforward neurovascular coupling and a viscoelastic effect on the Balloon model; (3) a BOLD signal change equation that accounts for magnetic field differences; and (4) stochastic (generalized filtering) model inversion that addresses non-linear interactions among hidden states, such as the non-linear increase in GABAergic inhibitory drive (Moran et al., 2011; Daunizeau et al., 2012) under anesthesia.

This paper presents the motivations for applying DCM to NHP fMRI and potential strategies for addressing anesthetic effects on neuronal activity and BOLD response, which is pertinent in primate neuroimaging under anesthesia. Clearly, a number of challenges remain. For example, the validity of this physiologic-stochastic DCM integration needs to be established. Additionally, its applicability to resting state data is also an interesting consideration. Stochastic DCM has been validated in resting-state fMRI (Razi et al., 2015); however, P-DCM has only been applied to task-based recordings (Havlicek et al., 2017). Future investigations are needed to examine the face and construct validity of IPS-DCM, as well as its applicability to resting-state data.

## Author Contributions

DJ and DD conceived the review focus, reviewed the literature, finalized the manuscript, and approved the final version of the manuscript. DJ summarized the literature review and wrote the first draft.

### Conflict of Interest Statement

The authors declare that the research was conducted in the absence of any commercial or financial relationships that could be construed as a potential conflict of interest.

## References

[B1] Adams D. L. Piserchia V. Economides J. R. Horton J. C. (2015). Vascular supply of the cerebral cortex is specialized for cell layers but not columns. Cereb. Cortex 25, 3673–3681. 10.1093/cercor/bhu221 25246513 PMC4585511

[B2] Aksenov D. P. Li L. Miller M. J. Iordanescu G. Wyrwicz A. M. (2015). Effects of anesthesia on BOLD signal and neuronal activity in the somatosensory cortex. J. Cereb. Blood Flow Metab. 35, 1819–1826. 10.1038/jcbfm.2015.130 26104288 PMC4635237

[B3] Attwell D. Buchan A. M. Charpak S. Lauritzen M. MacVicar B. Newman E. (2010). Glial and neuronal control of brain blood flow. Nature 468, 232–243. 10.1038/nature09613 21068832 PMC3206737

[B4] Attwell D. Iadecola C. (2002). The neural basis of functional brain imaging signals. Trends Neurosci. 25, 621–625. 10.1016/S0166-2236(02)02264-6 12446129

[B5] Bell M. A. Ball M. J. (1985). Laminar variation in the microvascular architecture of normal human visual cortex (area 17). Brain Res. 335, 139–143. 4005537 10.1016/0006-8993(85)90284-7

[B6] Bernal-Casas D. Balaguer-Ballester E. Gerchen M. F. Iglesias S. Walter H. Heinz A.. (2013). Multi-site reproducibility of prefrontal-hippocampal connectivity estimates by stochastic DCM. NeuroImage 82, 555–563. 10.1016/j.neuroimage.2013.05.120 23747286

[B7] Berwick J. Martin C. Martindale J. Jones M. Johnston D. Zheng Y.. (2002). Hemodynamic response in the unanesthetized rat: intrinsic optical imaging and spectroscopy of the barrel cortex. J. Cereb. Blood Flow Metab. 22, 670–679. 10.1097/00004647-200206000-00005 12045665

[B8] Bielczyk N. Z. Uithol S. van Mourik T. Anderson P. Glennon J. C. Buitelaar J. K. (2019). Disentangling causal webs in the brain using functional magnetic resonance imaging: A review of current approaches. Netw. Neurosci. 3, 237–273. 10.1162/netn_a_00062 30793082 PMC6370462

[B9] Boly M. Moran R. Murphy M. Boveroux P. Bruno M. A. Noirhomme Q.. (2012). Connectivity changes underlying spectral EEG changes during propofol-induced loss of consciousness. J. Neurosci. 32, 7082–7090. 10.1523/JNEUROSCI.3769-11.2012 22593076 PMC3366913

[B10] Brown G. G. Eyler Zorrilla L. T. Georgy B. Kindermann S. S. Wong E. C. Buxton R. B. (2003). BOLD and perfusion response to finger-thumb apposition after acetazolamide administration: differential relationship to global perfusion. J. Cereb. Blood Flow Metab. 23, 829–837. 10.1097/01.WCB.0000071887.63724.B2 12843786

[B11] Buijink A. W. G. van der Stouwe A. M. M. Broersma M. Sharifi S. Groot P. F. C. Speelman J. D.. (2015). Motor network disruption in essential tremor: a functional and effective connectivity study. Brain 138, 2934–2947. 10.1093/brain/awv225 26248468

[B12] Buxton R. B. Griffeth V. E. Simon A. B. Moradi F. Shmuel A. (2014). Variability of the coupling of blood flow and oxygen metabolism responses in the brain: a problem for interpreting BOLD studies but potentially a new window on the underlying neural activity. Front. Neurosci. 8:139. 10.3389/fnins.2014.00139 24966808 PMC4052822

[B13] Buxton R. B. Uludag K Dubowitz D. J. Liu T. T. (2004). Modeling the hemodynamic response to brain activation. Neuroimage 23 (Suppl. 1), S220–S233. 10.1016/j.neuroimage.2004.07.013 15501093

[B14] Buxton R. B. Wong E. C. Frank L. R. (1998). Dynamics of blood flow and oxygenation changes during brain activation: the balloon model. Magn. Reson. Med. 39, 855–864. 10.1002/mrm.1910390602 9621908

[B15] Chen J. J. Pike G. B. (2009). Origins of the BOLD post-stimulus undershoot. Neuroimage. 46, 559–568. 10.1016/j.neuroimage.2009.03.015 19303450

[B16] Cohen E. R. Ugurbil K. Kim S.-G. (2002). Effect of basal conditions on the magnitude and dynamics of the blood oxygenation level-dependent fMRI response. J. Cerebr. Blood Flow Metabol. 22, 1042–1053. 10.1097/00004647-200209000-00002 12218410

[B17] Daunizeau J. Friston K. Kiebel S. (2009). Variational Bayesian identification and prediction of stochastic nonlinear dynamic causal models. Phys. D 238, 2089–2118. 10.1016/j.physd.2009.08.002 19862351 PMC2767160

[B18] Daunizeau J. Stephan K. E. Friston K. J. (2012). Stochastic dynamic causal modelling of fMRI data: should we care about neural noise? Neuroimage 62, 464–481. 10.1016/j.neuroimage.2012.04.061 22579726 PMC3778887

[B19] Dehaene S. Piazza M. Pinel P. Cohen L. (2003). Three parietal circuits for number processing. Cogn. Neuropsychol. 20, 487–506. 10.1080/02643290244000239 20957581

[B20] Di X. Biswal B. B. (2014). Identifying the default mode network structure using dynamic causal modeling on resting-state functional magnetic resonance imaging. Neuroimage 86:53e59. 10.1016/j.neuroimage.2013.07.071 23927904 PMC3947265

[B21] Duncan J. Seitz R. J. Kolodny J. Bor D. Herzog H. Ahmed A.. (2000). A neural basis for general intelligence. Science 289, 457–460. 10.1126/science.289.5478.457 10903207

[B22] Duvernoy H. M. Delon S. Vannson J. L. (1981). Cortical blood vessels of the human brain. Brain Res. Bull. 7, 519–579. 10.1016/0361-9230(81)90007-1 7317796

[B23] Eichling J. O. Raichle M. E. Grubb R. L Jr. Larson K. B. Ter-Pogossian M. M. (1975). *In vivo* determination of cerebral blood volume with radioactive oxygen-15 in the monkey. Circ. Res. 37, 707–714. 10.1161/01.RES.37.6.707 811413

[B24] Frässle S. Lomakina E. I. Kasper L. Manjaly Z. M. Leffe A. Pruessmann K. P.. (2018). A generative model of whole-brain effective connectivity. NeuroImage 179, 505–529. 10.1016/j.neuroimage.2018.05.058 29807151

[B25] Frey S. H. (2008). Tool use, communicative gesture, and cerebral asymmetries in the modern human brain. Philos. Trans. R. Soc. Lond. B. Biol. Sci. 363, 1951–1957. 10.1098/rstb.2008.0008 18292060 PMC2606701

[B26] Friston K. (2009). Causal modelling and brain connectivity in functional magnetic resonance imaging. PLoS Biol. 7:e1000033. 10.1371/journal.pbio.1000033 19226186 PMC2642881

[B27] Friston K. Mattout J. Trujillo-Barreto N. Ashburner J. Penny W. (2007). Variational free energy and the Laplace approximation. Neuroimage 34, 220–234. 10.1016/j.neuroimage.2006.08.035 17055746

[B28] Friston K. J. (2011). Functional and effective connectivity: a review. Brain Connect. 1, 13–36. 10.1089/brain.2011.0008 22432952

[B29] Friston K. J. Glaser D. E. Henson R. N. Kiebel S. Phillips C. Ashburner J. (2002). Classical and Bayesian inference in neuroimaging: applications. Neuroimage 16, 484–512. 10.1006/nimg.2002.1091 12030833

[B30] Friston K. J. Harrison L. Penny W. (2003). Dynamic causal modelling. Neuroimage. 19, 1273–1302. 10.1016/S1053-8119(03)00202-7 12948688

[B31] Friston K. J. Kahan J. Biswal B. Razi A. (2014). A DCM for resting state fMRI. Neuroimage 94, 396–407. 10.1016/j.neuroimage.2013.12.009 24345387 PMC4073651

[B32] Friston K. J. Preller K. H. Mathys C. Cagnan H. Heinzle J. Razi A.. (2017). Dymamic causal modelling revisited. Neuroimage. 199, 730–744. 10.1016/j.neuroimage.2017.02.045 28219774 PMC6693530

[B33] Goebel R. Roebroeck A. Kim D. S. Formisano E. (2003). Investigating directed cortical interactions in time-resolved fMRI data using vector autoregressive modeling and Granger causality mapping. Magn. Reson. Imaging 21, 1251–1261. 10.1016/j.mri.2003.08.026 14725933

[B34] Gómez F. Phillips C. Soddu A. Boly M. Boveroux P. Vanhaudenhuyse A.. (2013). Changes in effective connectivity by propofol sedation. PLoS ONE 8:e71370. 10.1371/journal.pone.0071370 23977030 PMC3747149

[B35] Granger C. W. J. (1969). Investigating causal relations by econometric models and cross-spectral methods. Econometrica 37, 424–438. 10.2307/1912791

[B36] Grubb R. L Jr. Raichle M. E. Eichling J. O. Ter-Pogossian M. M. (1974). The effects of changes in PaCO_2_ on cerebral blood volume, blood flow, and vascular mean transit time. Stroke 5, 630–639. 10.1161/01.STR.5.5.630 4472361

[B37] Guibert R. Fonta C. Plouraboué F. (2010). Cerebral blood flow modeling in primate cortex. J. Cereb. Blood Flow Metab. 30, 1860–1873. 10.1038/jcbfm.2010.105 20648040 PMC3023927

[B38] Havlicek M. Ivanov D. Roebroeck A. Uludag K. (2017). Determining excitatory and inhibitory neuronal activity from multimodal fMRI data using a generative hemodynamic model. Front. Neurosci. 11:616. 10.3389/fnins.2017.00616 29249925 PMC5715391

[B39] Havlicek M. Roebroeck A. Friston K. Gardumi A. Ivanov D. Uludag K. (2015). Physiologically informed dynamic causal modeling of fMRI data. Neuroimage 122, 355–372. 10.1016/j.neuroimage.2015.07.078 26254113

[B40] Heinzle J. Koopmans P. J. den Ouden H. E. Raman S. Stephan K. E. (2016). A hemodynamic model for layered BOLD signals. Neuroimage 125, 556–570. 10.1016/j.neuroimage.2015.10.025 26484827

[B41] Heinzle J. Stephan K. (2018). Chapter 5: Dynamic causal modeling and its application to psychiatric disorders, in Computational Psychiatry, eds Anticevic A. Murray J. D. (Cambirdge, MA: Academic Press), 117–144.

[B42] Hillebrandt H. Friston K. J. Blakemore S. J. (2014). Effective connectivity during animacy perception–dynamic causal modelling of Human Connectome Project data. Sci. Rep. 4:6240. 10.1038/srep06240 25174814 PMC4150124

[B43] Hutchison R. M. Hutchison M. Manning K. Y. Menon R. S. Everling S. (2014). Isoflurane induces dose-dependent alterations in the cortical connectivity profiles and dynamic properties of the brain's functional architecture. Hum. Brain Mapp. 35, 5754–5775. 10.1002/hbm.22583 25044934 PMC6869297

[B44] Imas O. A. Ropella K. M. Ward B. D. Wood J. D. Hudetz A. G. (2005). Volatile anesthetics disrupt frontal-posterior recurrent information transfer at gamma frequencies in rat. Neurosci. Lett. 387, 145–150. 10.1016/j.neulet.2005.06.018 16019145

[B45] Ito H. Kanno I. Kato C. Sasaki T. Ishii K. Ouchi Y.. (2004). Database of normal human cerebral blood flow, cerebral blood volume, cerebral oxygen extraction fraction and cerebral metabolic rate of oxygen measured by positron emission tomography with 15O-labelled carbon dioxide or water, carbon monoxide and oxygen: a multicentre study in Japan. Eur. J. Nucl. Med. Mol. Imaging 31, 635–643. 10.1007/s00259-003-1430-8 14730405

[B46] Kahan J. Foltynie T. (2013). Understanding DCM: ten simple rules for the clinician. Neuroimage 83, 542–549. 10.1016/j.neuroimage.2013.07.008 23850463

[B47] Kang J. H. Choi J. H. Hwang E. Kim S. P. (2016). Changes in effective connectivity of sensorimotor rhythms in thalamocortical circuits during the induction and recovery of anesthesia in mice. J. Neurol. Sci. 369, 165–175. 10.1016/j.jns.2016.08.031 27653884

[B48] Kim P. J. Kim H. G. Noh G. J. Koo Y. S. Shin T. J. (2017). Disruption of frontal-parietal connectivity during conscious sedation by propofol administration. Neuroreport 28, 896–902. 10.1097/WNR.0000000000000853 28800575

[B49] Ku S. W. Lee U. Noh G. J. Jun I. G. Mashour G. A. (2011). Preferential inhibition of frontal-to-parietal feedback connectivity is a neurophysiologic correlate of general anesthesia in surgical patients. PLoS ONE 6:e25155. 10.1371/journal.pone.0025155 21998638 PMC3187752

[B50] Långsjö J. W. Maksimow A. Salmi E. Kaisti K. Aalto S. Oikonen V.. (2005). S-ketamine anesthesia increases cerebral blood flow in excess of the metabolic needs in humans. Anesthesiology 103, 258–268. 10.1097/00000542-200508000-00008 16052107

[B51] Lee U. Kim S. Noh G. J. Choi B. M. Hwang E. Mashour G. A. (2009). The directionality and functional organization of frontoparietal connectivity during consciousness and anesthesia in humans. Conscious. Cogn. 18, 1069–1078. 10.1016/j.concog.2009.04.004 19443244

[B52] Li B. Daunizeau J. Stephan K. E. Penny W. Hu D. Friston K. (2011). Generalised filtering and stochastic DCM for fMRI. Neuroimage 58, 442–457. 10.1016/j.neuroimage.2011.01.085 21310247

[B53] Li C.-X. Patel S. Auerbach E. J. Zhang X. (2013). Dose-dependent effect of isoflurane on regional cerebral blood flow in anesthetized macaque monkeys. Neurosci. Lett. 541, 58–62. 10.1016/j.neulet.2013.02.007 23428509 PMC4349366

[B54] Li L. Hu X. Preuss T. M. Glasser M. F. Damen F. W. Qiu Y.. (2013). Mapping putative hubs in human, chimpanzee and rhesus macaque connectomes via diffusion tractography. Neuroimage 80, 462–474. 10.1016/j.neuroimage.2013.04.024 23603286 PMC3720835

[B55] Li T.-Q. Kastrup A. Moseley M. E. Glover G. H. (2000). Changes in baseline cerebral blood flow in humans do not influence regional cerebral blood flow response to photic stimulation. J. Magn. Reson. Imaging 12, 757–762. 10.1002/1522-2586(200011)1 2:53.0.CO;2-4 11050647

[B56] Lindauer U. Leithner C. Kaasch H. Rohrer B. Foddis M. Füchtemeier M.. (2010). Neurovascular coupling in rat brain operates independent of hemoglobin deoxygenation. J. Cereb Blood Flow Metab. 30, 757–768. 10.1038/jcbfm.2009.259 20040927 PMC2949158

[B57] Lohmann G. Erfurth K. Muller K. Turner R. (2012). Critical comments on dynamic causal modelling. Neuroimage 59, 2322–2329. 10.1016/j.neuroimage.2011.09.025 22001162

[B58] MacVicar B. A. Newman E. A. (2015). Astrocyte regulation of blood flow in the brain. Cold Spring Harb Perspect. Biol. 7:a020388. 10.1101/cshperspect.a020388 25818565 PMC4448617

[B59] Mandeville J. B. Marota J. J. Ayata C. Zararchuk G. Moskowitz M. A. Rosen B.. (1999). Evidence of a cerebrovascular postarteriole Windkessel with delayed compliance. J. Cereb. Blood Flow Metab. 19, 679–689. 10.1097/00004647-199906000-00012 10366199

[B60] Mantini D. Corbetta M. Romani G. L. Orban G. A. Vanduffel W. (2013). Evolutionarily novel functional networks in the human brain? J. Neurosci. 33, 3259–3275. 10.1523/JNEUROSCI.4392-12.2013 23426655 PMC6619535

[B61] Marreiros A. C. Kiebel S. J. Friston K. J. (2008). Dynamic causal modelling for fMRI: a two-state model. Neuroimage 39, 269–278. 10.1016/j.neuroimage.2007.08.019 17936017

[B62] Mars R. B. Jbabdi S. Sallet J. O'Reilly J. X. Croxson P. L. Olivier E.. (2011). Diffusion-weighted imaging tractography-based parcellation of the human parietal cortex and comparison with human and macaque resting-state functional connectivity. J. Neurosci. 31, 4087–4100. 10.1523/JNEUROSCI.5102-10.2011 21411650 PMC3091022

[B63] Martin C. Martindale J. Berwick J. Mayhew J. (2006). Investigating neural-hemodynamic coupling and the hemodynamic response function in the awake rat. Neuroimage 32, 33–48. 10.1016/j.neuroimage.2006.02.021 16725349

[B64] Masamoto K. Kim T. Fukuda M. Wang P. Kim S. G. (2007). Relationship between neural, vascular, and BOLD signals in isoflurane-anesthetized rat somatosensory cortex. Cereb. Cortex 17, 942–950. 10.1093/cercor/bhl005 16731882

[B65] Moran R. J. Jung F. Kumagai T. Endepols H. Graf R. Dolan R. J.. (2011). Dynamic causal models and physiological inference: a validation study using isoflurane anaesthesia in rodents. PLoS ONE 6:e22790. 10.1371/journal.pone.0022790 21829652 PMC3149050

[B66] Ogawa S. Tank D. W. Menon R. Ellermann J. M. Kim S. G. Merkle H.. (1992). Intrinsic signal changes accompanying sensory stimulation: functional brain mapping with magnetic resonance imaging. Proc. Natl. Acad. Sci. U.S.A. 89:5951–5955. 10.1073/pnas.89.13.5951 1631079 PMC402116

[B67] Olsen K. S. Henriksen L. Owen-Falkenberg A. Dige-Petersen H. Rosenorn J. Chraemmer-Jorgensen B. (1994). Effect of 1 or 2 MAC isoflurane with or without ketanserin on cerebral blood flow autoregulation in man. Br. J. Anaesth. 72, 66–71. 10.1093/bja/72.1.66 8110555

[B68] Paasonen J. Stenroos P. Salo R. A. Kiviniemi V. Gröhn O. (2018). Functional connectivity under six anesthesia protocols and the awake condition in rat brain. Neuroimage 172, 9–20. 10.1016/j.neuroimage.2018.01.014 29414498

[B69] Park H. J. Friston K. J. Pae C. Park B. Razi A. (2018). Dynamic effective connectivity in resting state fMRI. Neuroimage 180(Pt B), 594–608. 10.1016/j.neuroimage.2017.11.033 29158202 PMC6138953

[B70] Pernet C. R. (2014). Misconceptions in the use of the General Linear Model applied to functional MRI: a tutorial for junior neuro-imagers. Front. Neurosci. 8:1. 10.3389/fnins.2014.00001 24478622 PMC3896880

[B71] Phelps M. E. Grubb R. L Jr. Ter-Pogossian M. M. (1973). *In vivo* regional cerebral blood volume by x-ray fluorescence: validation of method. J. Appl. Physiol. 35, 741–747. 10.1152/jappl.1973.35.5.741 4203706

[B72] Powers W. J. Hirsch I. B. Cryer P. E. (1996). Effect of stepped hypoglycemia on regional cerebral blood flow response to physiological brain activation. Am. J. Physiol. 270, H554–H559. 10.1152/ajpheart.1996.270.2.H554 8779830

[B73] Razi A. Kahan J. Rees G. Friston K. J. (2015). Construct validation of a DCM for resting state fMRI. Neuroimage 106, 1–14. 10.1016/j.neuroimage.2014.11.027 25463471 PMC4295921

[B74] Roebroeck A. Formisano E. Goebel R. (2005). Mapping directed influence over the brain using Granger causality and fMRI. Neuroimage 25, 230–242. 10.1016/j.neuroimage.2004.11.017 15734358

[B75] Rowe J. B. Hughes L. E. Barker R. A. Owen A. M. (2010). Dynamic causal modelling of effective connectivity from fMRI: are results reproducible and sensitive to Parkinson's disease and its treatment? Neuroimage 52, 1015–1026. 10.1016/j.neuroimage.2009.12.080 20056151 PMC3021391

[B76] Sallet J. Mars R. B. Noonan M. P. Neubert F.-X. Jbabdi S. O'Reilly J. X.. (2013). The organization of dorsal frontal cortex in humans and macaques. J. Neurosci. 33, 12255–12274. 10.1523/JNEUROSCI.5108-12.2013 23884933 PMC3744647

[B77] Scharrer E. (1960). Brain Function and the Evolution of Cerebral Vascularization. James Arthur Lecture on the Evolution of the Human Brain. New York: The American Museum of Natural History, f−32.

[B78] Schreiber T. (2000). Measuring information transfer. Phys. Rev. Lett. 85, 461–464. 10.1103/PhysRevLett.85.461 10991308

[B79] Schuyler B. Ollinger J. M. Oakes T. R. Johnstone T. Davidson R. J. (2010). Dynamic causal modeling applied to fMRI data shows high reliability. Neuroimage 49, 603–611. 10.1016/j.neuroimage.2009.07.015 19619665 PMC2867050

[B80] Seth A. K. Barrett A. B. Barnett L. (2015). Granger causality analysis in neuroscience and neuroimaging. J. Neurosci. (2015) 35, 3293–3297. 10.1523/JNEUROSCI.4399-14.2015 25716830 PMC4339347

[B81] Singer T. Lamm C. (2009). The social neuroscience of empathy. Ann. N. Y. Acad. Sci. 1156, 81–96. 10.1111/j.1749-6632.2009.04418.x 19338504

[B82] Slupe A. M. Kirsch J. R. (2018). Effects of anesthesia on cerebral blood flow, metabolism, and neuroprotection. J. Cereb. Blood Flow Metabol. 38, 2192–2208. 10.1177/0271678X18789273 30009645 PMC6282215

[B83] Smith S. Miller K. Salimi-Khorshidi G. Webster M. Beckmann C. Nichols T. Woolrich M. (2011). Network modelling methods for fMRI. NeuroImage 54, 875–891. 10.1016/j.neuroimage.2010.08.063 20817103

[B84] Stephan K. E. Harrison L. M. Penny W. D. Friston K. J. (2004). Biophysical models of fMRI responses. Curr. Opin. Neurobiol. 14, 629–635. 10.1016/j.conb.2004.08.006 15464897

[B85] Stephan K. E. Penny W. D. Moran R. J. den Ouden H. E. Daunizeau J. Friston K. J. (2010). Ten simple rules for dynamic causal modeling. Neuroimage 49, 3099–3109. 10.1016/j.neuroimage.2009.11.015 19914382 PMC2825373

[B86] Stephan K. E. Weiskopf N. Drysdale P. M. Robinson P. A. Friston K. J. (2007). Comparing hemodynamic models with DCM. Neuroimage 38, 387–401. 10.1016/j.neuroimage.2007.07.040 17884583 PMC2636182

[B87] Tak S. Noh J. Cheong C. Zeidman P. Razi A. Penny W. D.. (2018). A validation of dynamic causal modelling for 7T fMRI. J. Neurosci. Methods 305, 36–45. 10.1016/j.jneumeth.2018.05.002 29758234

[B88] Uludag K. Müller-Bierl B. Ugurbil K. (2009). An integrative model for neuronal activity-induced signal changes for gradient and spin echo functional imaging. Neuroimage 48, 150–165. 10.1016/j.neuroimage.2009.05.051 19481163

[B89] Van Aken H. Fitch W. Graham D. I. Brussel T. Themann H. (1986). Cardiovascular and cerebrovascular effects of isoflurane-induced hypotension in the baboon. Anesth. Analg. 65, 565–574. 10.1213/00000539-198606000-00003 3706797

[B90] Van Aken H. van Hemelrijck J. (1991). Influence of anesthesia on cerebral blood flow and cerebral metabolism: an overview. Agressologie 32, 303–306. 1843831

[B91] Van Essen D. C. Dierker D. L. (2007). Surface-based and probabilistic atlases of primate cerebral cortex. Neuron 56, 209–225. 10.1016/j.neuron.2007.10.015 17964241

[B92] Vaudano A. E. Avanzini P. Tassi L. Ruggieri A. Cantalupo G. Benuzzi F. … Meletti S (2013). Causality within the epileptic network: an EEG-fMRI study validated by intracranial EEG. Front. Neurol. 14:185 10.3389/fneur.2013.00185 PMC3827676 24294210

[B93] Vicente R. Wibral M. Lindner M. Pipa G. (2011). Transfer entropy—a model-free measure of effective connectivity for the neurosciences. J. Comput. Neurosci. 30, 45–67. 10.1007/s10827-010-0262-3 20706781 PMC3040354

[B94] Webb J. T. Ferguson M. A. Nielsen J. A. Anderson J. S. (2013). BOLD Granger causality reflects vascular anatomy. PLoS ONE 8:e84279. 10.1371/journal.pone.0084279 24349569 PMC3862772

[B95] Weber B. Keller A. L. Reichold J. Logothetis N. K. (2008). The microvascular system of the striate and extrastriate visual cortex of the macaque. Cereb. Cortex. 18:2318–2330. 10.1093/cercor/bhm259 18222935

[B96] White N. S. Alkire M. T. (2003). Impaired thalamocortical connectivity in humans during general-anesthetic-induced unconsciousness. Neuroimage 19(2 Pt 1), 402–411. 10.1016/S1053-8119(03)00103-4 12814589

[B97] Witt S. T. Meyerand M. E. (2009). The effects of computational method, data modeling, and TR on effective connectivity results. Brain Imaging Behav. 3, 220–231. 10.1007/s11682-009-9064-5 19714255 PMC2731943

[B98] Wright S. (1920). The relative importance of heredity and environment in determining the piebald pattern of guinea-pigs. Proc. Natl. Acad. Sci. U.S.A. 6, 320–332. 10.1073/pnas.6.6.320 16576506 PMC1084532

[B99] Yeom S. K. Won D. O. Chi S. I. Seo K. S. Kim H. J. Müller K. R.. (2017). Spatio-temporal dynamics of multimodal EEG-fNIRS signals in the loss and recovery of consciousness under sedation using midazolam and propofol. PLoS ONE 12:e0187743. 10.1371/journal.pone.0187743 29121108 PMC5679575

[B100] Zappe A. C. Uludag K. Oeltermann A. Ugurbil K. Logothetis N. K. (2008). The influence of moderate hypercapnia on neural activity in the anesthetized non-human primate. Cereb. Cortex 18, 2666–2673. 10.1093/cercor/bhn023 18326521 PMC2567427

